# Twitter Users’ Views on Mental Health Crisis Resolution Team Care Compared With Stakeholder Interviews and Focus Groups: Qualitative Analysis

**DOI:** 10.2196/25742

**Published:** 2021-06-29

**Authors:** Natasha Chilman, Nicola Morant, Brynmor Lloyd-Evans, Jane Wackett, Sonia Johnson

**Affiliations:** 1 Division of Psychiatry University College London London United Kingdom; 2 Department of Psychological Medicine Institute of Psychiatry, Psychology & Neuroscience King's College London London United Kingdom; 3 Camden and Islington NHS Foundation Trust London United Kingdom

**Keywords:** Twitter, social media, qualitative, crisis resolution team, home treatment team, mental health, acute care, severe mental illness

## Abstract

**Background:**

Analyzing Twitter posts enables rapid access to how issues and experiences are socially shared and constructed among communities of health service users and providers, in ways that traditional qualitative methods may not.

**Objective:**

To enrich the understanding of mental health crisis care in the United Kingdom, this study explores views on crisis resolution teams (CRTs) expressed on Twitter. We aim to identify the similarities and differences among views expressed on Twitter compared with interviews and focus groups.

**Methods:**

We used Twitter’s advanced search function to retrieve public tweets on CRTs. A thematic analysis was conducted on 500 randomly selected tweets. The principles of refutational synthesis were applied to compare themes with those identified in a multicenter qualitative interview study.

**Results:**

The most popular hashtag identified was *#CrisisTeamFail*, where posts were principally related to poor quality of care and access, particularly for people given a *personality disorder* diagnosis. Posts about CRTs giving unhelpful self-management advice were common, as were tweets about resource strains on mental health services. This was not identified in the research interviews. Although each source yielded unique themes, there were some overlaps with themes identified via interviews and focus groups, including the importance of rapid access to care. Views expressed on Twitter were generally more critical than those obtained via face-to-face methods.

**Conclusions:**

Traditional qualitative studies may underrepresent the views of more critical stakeholders by collecting data from participants accessed via mental health services. Research on social media content can complement traditional or face-to-face methods and ensure that a broad spectrum of viewpoints can inform service development and policy.

## Introduction

### Twitter in Mental Health Research

Twitter has emerged as a prominent social media platform that may be a valuable data source for researchers wanting to access stakeholders’ views on many topics, including mental health services [1]. Results from a survey of mental health professionals suggest that psychiatrists and psychologists hold mixed views on using social media in their professional lives [2]. However, in recent years, researchers have used Twitter as a source of qualitative data to explore mental health by analyzing tweets from the general public regarding depression [3-7], schizophrenia [7,8], Alzheimer disease [9], suicide [10], stigma [11], and mental health professional conduct [1]. This research has found active communities of Twitter users discussing topics related to mental health and mental health care [5].

Twitter has its own methodological strengths and limitations as a source of secondary data for research. First, it offers researchers a quick and affordable way to access textual data that can be analyzed both qualitatively and quantitatively. For example, researchers have analyzed Twitter posts to provide rapid insights into the impact of the COVID-19 pandemic [12-14]. Twitter allows information to be collected in a naturalistic setting and therefore captures meaningful moments for Twitter users, which are less subject to the researcher’s influence [4,15,16]. However, Twitter analysis clearly only accesses the views of those who use the internet and engage with this particular social media platform. Tweets are currently more likely to be from younger, more affluent sectors of the population [17,18]. Twitter users may sometimes tweet in a *reactive* way, conveying *in-the-moment* feelings and experiences [19] rather than more reflective views. Finally, short tweets can lack depth of content and context, making them harder to make inferences from when compared with rich and complex interview transcripts [5]. Similar to any data collection method, these strengths and limitations are important to consider when analyzing Twitter data in mental health research.

Research using Twitter or other social media data relies on the premise that the data reflect authentic experiences. Recently, commentators have attempted to evaluate the authenticity of Twitter posts [20-22]. Twitter has a public declarative function, which might influence the way people communicate on the platform and the content they share [23]. Social media platforms provide a forum for the construction and curation of social identities via a rapid exchange of personally, socially, and politically relevant information [23]. As such, data obtained via this medium need to be understood within the context of these multiple agendas [24,25]. Nonetheless, Twitter has been viewed as an extension of the real world [26,27]. Where Twitter posts are considered within their context, they can provide researchers with authentic knowledge of people’s daily rituals, opinions, and experiences [23].

Traditional qualitative methods are usually labor-intensive and costly [28]. In research environments where resources are scarce and where rapid investigations of stakeholder views with direct clinical and policy relevance are needed, Twitter may be a readily accessible opportunity for data collection. Twitter may also provide novel perspectives on mental health service provision, as users posting on social media platforms may be different from those who agree to participate in research, especially research in which participants are recruited via mental health services. However, studies have not yet investigated how views on mental health care are expressed on Twitter compared with those identified through traditional qualitative data collection methods, and little is known about the unique contribution of a Twitter analysis.

### Crisis Resolution Teams

Crisis resolution teams (CRTs; sometimes known as home treatment teams) were implemented nationally in the United Kingdom following policy directives in the National Health Service (NHS) in 2000 [29]. The remit of CRTs is to provide intensive community support to those experiencing a mental health crisis, with the aim of reducing the need for inpatient psychiatric hospital admission [30,31]. Research using electronic health record data indicates that most patients using CRT services are White, unmarried, middle-aged, and live in areas of high social deprivation [32]. Although research has shown that CRT support increases service user satisfaction with acute care in some circumstances [31,33,34], this is not consistent across services [35,36]. Furthermore, CRTs have not reduced admission rates nationwide to the degree anticipated [32,37]. Stakeholder perspectives of CRT care can inform initiatives to improve implementation, service user experiences, and outcomes [35,38]. The largest qualitative study on UK stakeholders’ views of CRTs to date was conducted by Morant et al [38] as part of the work to develop a model of CRT good practices [39]. The thematic analysis found that staff continuity, carer involvement, and emotional and practical support are important components for CRT implementation [38].

### Study Aims

Combining different sources for qualitative analyses is advocated to achieve a comprehensive understanding of a phenomenon [40,41]. Thus far, research on CRTs has relied on traditional qualitative methods. In this study, we aim to explore Twitter posts about CRTs and compare these posts with views obtained via interviews and focus groups. We seek to extend our understanding of the potential of Twitter as an easily accessible source of relevant stakeholder views, compare findings from Twitter with those from more traditional qualitative methods, and consider what this adds to our understanding of CRT implementation as experienced by stakeholders, including service users, family carers, service providers, and mental health practitioners.

A common methodological pitfall when comparing and combining different qualitative methods is to claim that one method is *better* than another [40]. By comparing Twitter posts with data collected via face-to-face methods, we do not wish to verify one data collection method or claim that one method is more trustworthy; rather, we are interested in comparing findings from these data sources to explore similarities and differences among views expressed regarding CRTs in both contexts.

## Methods

### Ethics Approval and Consent to Participate

This study was designed in accordance with guidelines on social media research published by the British Psychological Society [42] and the Association of Internet Researchers [43] and was approved by the University College London ethics chair (project ID: 1329/001). As this study only used publicly available tweets, there was no requirement for consent to participate. Twitter quotes used in this study have been paraphrased. Paraphrased quotes were tested through Twitter and Google search engines to ensure that the identity of the Twitter users was protected. Ethical approval for the Crisis Team Optimization and Relapse Prevention (CORE) study was obtained from the London Camden and Islington Research Ethics Committee (REC reference number 10/H0722/84), and written informed consent was obtained from all study participants.

### Data Collection

#### Twitter

Twitter’s advanced search function was used to retrieve public tweets relevant to CRTs. Search terms were chosen through informal explorations on Twitter and included known terminology relevant to CRTs (eg, *home treatment team*) and relevant *hashtags* (including *#crisisteamfail* and *#crisisteamsuccess*). A full list of search terms is presented in Table 1.

**Table 1:** Total number of tweets retrieved, excluded, and randomly selected for analysis by the search term^a^.

Search term	Total retrieved tweets, n (%)	Date range of retrieved tweets	Excluded tweets^b^, n (%)	Included tweets, n (%)	Tweets randomly selected for analysis, n (%)
#CRHTT^c^	16 (0.33)	August 23, 2012-September 5, 2017	0 (0)	16 (0.38)	1 (0.2)
#crisisteam	516 (10.65)	December 6, 2011-May 25, 2018	109 (17.03)	407 (9.68)	186 (37.2)
#crisisteamfail	934 (19.27)	November 20, 2014-May 12, 2018	4 (0.63)	930 (22.11)	127 (25.4)
#crisisteamsuccess	26 (0.54)	November 20, 2015-November 25, 2017	0 (0)	26 (0.62)	6 (1.2)
#dearcrisisteam	147 (3.03)	November 24, 2014-May 1, 2018	0 (0)	147 (3.5)	30 (6)
#hometreatmentteam	20 (0.41)	September 10, 2013-May 29, 2018	1 (0.16)	19 (0.45)	1 (0.2)
Crisis resolution team	132 (2.72)	September 22, 2010-June 1, 2018	21 (3.28)	111 (2.64)	10 (2)
Home treatment team	1714 (35.37)	July 12, 2010-June 1, 2018	37 (5.78)	1677 (39.87)	209 (41.8)
Crisis team	1255 (25.9)	November 1, 2017-November 30, 2017; February 1, 2018-February 28, 2018	465 (72.66)	790 (18.78)	90 (18)
Total	4846 (100)	July 12, 2010-June 1, 2018	640 (100)	4206 (100)	500 (100)

^a^Some tweets were captured by more than one search term.

^b^Reasons for exclusion: tweet not clearly relevant to UK mental health crisis resolution teams, tweet not in the English language, or tweet suspected to be from or about minors.

^c^CRHTT: Crisis Resolution Home Treatment Team.

We collected tweets posted between January 1, 2010, and June 1, 2018. While conducting the search, we found that the term *crisis team* produced many results. Most of these tweets were irrelevant to the study’s focus on mental health CRTs. To ensure that the data set was manageable and relevant to the study, we collected 2 months of tweets for the *crisis team* search only. We assigned a number to each month in the overall period (January 1, 2010-June 1, 2018), then used a random number generator that randomly selected 2 months of tweets (November 2017 and February 2018) for the *crisis team* search. For all other search terms, we collected tweets posted between January 1, 2010, and June 1, 2018.

As tweets were extracted into an encrypted Microsoft Excel document, identifying features such as usernames and pictures were removed. Tweets were included if they were clearly relevant to UK mental health CRTs and excluded if they were not in the English language or were suspected to be from or about minors. The latter included tweets that either mentioned Child and Adolescent Mental Health Services or explicitly stated that their or someone else’s age was below 16 years. We did not analyze the number of retweets or responses. Tweets citing external links were included, but these links were not accessed or analyzed.

#### CORE Interviews and Focus Groups

We used interview and focus group data from the CORE study for a comparison with the Twitter data. CORE was a research program that aimed to define and improve fidelity to good practice in mental health crisis teams [44]. A qualitative study was conducted as a part of this, aiming to explore stakeholders’ experiences and views of CRTs and identify what they considered important for good CRT care. Semistructured interviews and focus groups were conducted with service users (n=41), carers (n=20), and CRT practitioners (n=137). Participants were recruited via CRT clinicians from 10 mental health catchment areas in England. The topics covered included experiences of CRT care, access to and discharge from CRTs, what is most important in CRT care, suggestions for best practices, and any barriers to or facilitators of achieving good practice. Questions were broad and open to allow respondents to bring up what they found important. Further details can be found in the previous study [38]. In this study, researchers had access to the full set of anonymized transcripts as a secondary data source.

### Data Analysis

#### Twitter Analysis

Once all search outputs had been collated, tweets were selected for analysis using a random number generator. Tweets were selected and analyzed, 100 tweets at a time. Tweets were organized into basic descriptive categories following the principles of conventional content analysis [45] to provide an overview of the data set. If the tweet stated what kind of stakeholder was tweeting, for example, a service user or staff member, this was recorded as the tweet *relation*.

The main qualitative analysis of the Twitter data used thematic analysis, following the guidelines by Braun and Clarke [46,47]. We used an inductive approach to explore the data, although we were also guided by the awareness of findings from the previous CORE study. The lead researcher (NC) generated codes from the first 100 tweets to identify the initial themes. A second researcher (JW), who had lived experience of mental health service use, also conducted a thematic analysis on the first 100 tweets [48]. The 2 researchers discussed the tweets and combined their codes to collaboratively develop ideas on initial themes. The lead researcher then progressed through the data set of 100 tweets at a time, continuing with an inductive approach and transforming the coding frame as appropriate. One tweet could have multiple codes or contribute to multiple themes, although due to the short nature of tweets, this rarely occurred. The analysis was a collaborative and iterative process in which the wider research team met regularly to discuss the development of the coding frame and the clustering of codes into more abstract themes, and any uncertainties or disagreements between members of the team were resolved through discussion. The research team included various perspectives on the research topic, including lived experience, clinical roles, and academics working in CRT research. This iterative analysis process continued until thematic saturation was reached at 500 tweets. Changes or trends over time were not analyzed.

#### CORE Analysis

The CORE interviews and focus groups had already been analyzed using inductive thematic analysis by researchers in the original study [38]. The researchers in this study had access to the analyzed transcripts, notes, and coding frames on NVivo (QSR International). The lead researcher (NC) read this content to familiarize herself with the data and their interpretation in the original study. Other members of the research team worked on the original CORE study (NM, BLE, and SJ).

#### Comparative Analysis

The principles of refutational synthesis were used to compare the CORE qualitative interview data set to the Twitter data set. Refutational synthesis is a form of meta-ethnography that involves exploring and explaining contradictions among qualitative studies, with a focus on contextual differences [49,50]. We created tables, to structure the refutational synthesis, in which codes and themes from both data sets were listed against one another if they had any relation or contrast. Discussions on these comparisons were held by the research team.

## Results

### Twitter Search Results

Table 1 shows the number of tweets retrieved and included per search term. In total, 4206 tweets were retrieved from nine search terms. The highest number of relevant tweets was collected using the search term *home treatment team*. Hashtags identified in the search process included trending topics such as *#crisisteamfail*, which was the most frequently tweeted hashtag search term.

### Who Is Tweeting?

Owing to the search strategy and the nature of Twitter, we were unable to determine the precise demographics of our sample, such as age, location, or mental health diagnosis (if any) of *tweeters*. As the search was made from a computer in London, tweets are more likely (but not verifiably) to be from the United Kingdom. It was feasible to identify what kind of CRT stakeholder the Twitter user was with reasonable confidence for most analyzed tweets (381/500, 76.2%). Table 2 shows the total number and percentages of stakeholder groups for the 500 analyzed tweets. Service users were the most common group who tweeted about CRTs (239/500, 47.8%) with fewer tweets posted by job advertisers, staff, and family or friends. There was 87% agreement between the 2 coders for 100 tweet relations, indicating that it is feasible to identify who is tweeting consistently.

**Table 2:** Twitter user relation groups (who was tweeting) for analyzed tweets (N=500).

Relation group	Tweets, n (%)
Service user	239 (47.8)
Unknown	119 (23.7)
Job advertisers	60 (12)
Other	30 (6)
Family or friend	26 (5.2)
CRT^a^ staff	26 (5.2)

^a^CRT: crisis resolution team.

### What Do People Tweet About?

The content analysis for 500 tweets indicated that large proportion of tweets expressed negative views about CRTs (205/500, 41%), compared with a small proportion of posts about positive experiences (36/500, 7.2%). The remainder of tweets were more neutral, for example, personal updates. These personal updates involved the Twitter user sharing live updates on their whereabouts and activities, such as “I’m off to see the CRT.” Information sharing (80/500, 16%) was more common than information seeking (21/500, 4%). For example, Twitter users shared information on how to contact the CRT. A proportion of tweets included job advertisements to work in CRTs (62/500, 12.4%). Sometimes we could identify where *tweeters* were engaged in conversation with one another through the “@” symbol in their tweets (80/500, 16%), where they shared their experiences of CRT services and at times offered emotional support.

### Thematic Analysis of Twitter Content

We identified principally critical Twitter content about experiences of accessing CRTs and the quality of care provided, comments on resource limitations, and a small number of more positively toned tweets by CRT staff members.

#### Accessing Crisis Team Care

Among the critical posts, some Twitter users reported having difficulty accessing CRT care for themselves or a family member or friend. Twitter users described barriers to access help, such as feeling prematurely *turned away* by the CRT. This was particularly the case in which Twitter users posted that they had been diagnosed with a *personality disorder*. Other mental health diagnoses were rarely mentioned and were not associated with this experience of rejection from the CRT. Twitter users also reported difficulties in contacting the CRT, particularly by phone:

When the CRT find out you have a diagnosis of BPD, they just ignore you.

I’m lucky to have access to the crisis team—not everyone does just because of their diagnosis.

Just tried to ring the crisis team, no-one answered, no answerphone.

Although not all tweets discussing access to crisis care were negative, positive sentiments were sometimes tinged with surprise or sarcasm, suggesting a mixed landscape of experiences and a lack of consistent access to the CRT:

Crisis team staff came to see me the same day that I called them, what kind of parallel universe is this?

#### Quality of Care

Twitter users expressed varying experiences of quality of care, although sentiments were once again weighted toward the negative. There were many tweets about CRT staff giving inappropriately basic self-management advice to service users, such as having a cup of tea or taking a bath. Service users tweeted that, as a consequence, they felt that services did not understand or appreciate the severity of their crisis and that their own self-management strategies may have already been exhausted. They expressed finding distraction or self-care advice to be patronizing and insensitive. Discussions on inappropriate self-management advice have been so widely circulated on Twitter that, at times, Twitter users interacted with each other in tones of sarcasm:

Crisis team, if I’m talking to you I’ve already tried drinking tea and doing mindfulness colouring whilst in the bath.

A paramedic would never advise a patient to just go for a walk or watch TV, so why does the crisis team?

Of the smaller number of service user tweets about positive experiences of CRTs, these were often attributed to CRT staff listening to service users and providing meaningful, appropriate advice. Interestingly, Twitter users tended to attribute *success stories* to individual staff members. In other words, *#crisisteamsuccess* depended on the expertise of individual staff members:

There are some really excellent CRT staff members out there, they are rare but real.

The woman who visited from the crisis team this morning was so reassuring.

#### Strain on Mental Health Services

There were recurrent mentions of NHS cuts, a lack of hospital beds, and the use of police and paramedics to cover what was felt to be a lack of mental health professionals in the community. Such frustrations with the *system* were expressed by service users, family, and friends alike but were not mentioned by any self-identified CRT staff members in this sample:

The mental health system is in meltdown, further cuts mean services can’t cope, no hospital beds, crisis team overwhelmed, they need more funds.

My local CRT have no resources, no training, the system is broken.

#### Working in a CRT

A small number of Twitter users (26/500, 5.2%) were identified as CRT staff members. Although there were some suggestions for long and unsociable working hours, there was a positive trend in these tweets in which staff members expressed gratitude to their team. Most of these tweets seemed to be from nursing or medical trainees on placements, reflecting that Twitter is often used by a younger age group who may be more open to talking about their work on social media [2]:

Am sad that my time working in the CRT is coming to an end, it is a v good team #rewarding

Working on new years eve, yay for me! #mentalhealthcrisisteam

### Refutational Synthesis: Themes From Twitter Compared With Interviews and Focus Groups

#### Overview

The CORE study identified three domains that were important to stakeholders in CRT care: (1) the organization of CRT care (including easy and quick access to care and staff continuity), (2) the content of CRT work (including the staff being empathetic and providing emotional support), and (3) the role of CRTs in the wider system (eg, gatekeeping admissions). This was explored in detail in the original study publication, along with the demographics of the sample [38].

In the refutational synthesis, we found that the features identified as important for good access to and quality of CRT care in the CORE study aligned with the views of tweeters. However, there were differing views on how far this was currently being achieved in practice, with tweeters being generally more critical of CRT care. In Table 3, we list some of the comparisons among themes that highlight similarities and differences between the data sets. Here, we provide details on the areas of convergence and divergence in stakeholder views of the access and quality of CRT care. We also discuss the strain on mental health services as a theme that is unique to the Twitter data set.

**Table 3:** Results from the refutational synthesis examining similarities and differences among themes identified in the Twitter data set and the Crisis Team Optimization and Relapse Prevention interview and focus groups data sets, with code examples^a^.

Theme	Twitter data set	Interviews and focus groups data sets
**Accessing CRT^b^ care**		
	*Importance of quick and easy access*	*Importance of quick and easy access*
	*Reports of difficulty accessing the CRT, for example, via the phone*	—^c^
	*Diagnosis of a “personality disorder” as a barrier to accessing CRT care*	—
**Quality of CRT care**		
	*Importance of empathetic staff members*	*Importance of empathetic staff members*
	*Feeling dismissed by inappropriate distraction or self-care self-management advice*	—
	—	*Holistic models of care*
**Strain on mental health services**		
	*Underresourced community crisis services*	—
	*Use of police or paramedics in mental health crises*	—
**CRT’s role in the wider system**		
	—	*Gatekeeping hospital admissions*
	—	*CRT as an alternative to hospital*

^a^Codes are indicated using italics.

^b^CRT: crisis resolution team.

^c^Not identified.

#### Accessing CRT Care

The importance of having readily accessible crisis care was evident in both the CORE interviews and the Twitter data set. However, the two data sets illustrated very different service user experiences. As discussed, service users on Twitter largely complained that the CRT was not readily available, for example, by not answering the phone or being too far away. Such tweets were often in real time, reporting contact (or a lack of contact) with the CRT as it happened:

No one will answer the phone during handover for an hour, what if it’s an emergency?

Twitter user

In contrast to the largely negative views expressed on Twitter, CORE service users reported mixed experiences with access to care. Some service users reported that it was mostly quick and easy to contact the CRT by phone, and many appreciated that care was available during unsociable hours:

Overall, what matters most? I would say the fact that you can make contact with the Crisis team 24 hours, 24/7.

CORE service user

An important finding in the Twitter data set was that people who were diagnosed with a *personality disorder* felt that their diagnosis was a barrier to access care, and these Twitter users often felt that stigma against their diagnosis meant that they were denied care:

Crisis team said “CRT care for people with a diagnosis of BPD can make them worse, so we don’t really visit them.”

Twitter user

Issues regarding access to care for people with *personality disorders* did not arise in the CORE study. Among the small subset of service users in the CORE study with *personality disorder* diagnoses, the sense of rejection identified in the Twitter posts was not expressed. Conversely, these service users often reported positive service experiences and did not identify their diagnosis as a barrier to treatment. In focus groups with CRT staff members, there were diverging opinions regarding whether CRT support was appropriate for those with *personality disorder* diagnoses. Some staff expressed perceived difficulties in supporting people with these diagnoses, including difficulties in maintaining the boundaries of therapeutic relationships, difficulties with the time-limited nature of CRT support, and general pressures on mental health systems and teams:

More and more, we’ve got a lot of people on our books with borderline personality disorder...They refer to us because they [referrers] don’t know what to do with them, which is fine, but neither do we.

CORE CRT staff

In summary, although both data sets show a mixed landscape of experiences, Twitter users often reported more difficulty accessing CRT care. This was particularly the case for Twitter users who were posting about *personality disorder*.

#### Quality of Care

Both service users and carers in the CORE study reported mixed experiences of the quality of care received from CRTs. In the CORE interviews, help with practical issues, emotional support, and relationship building were all described as important aspects of good quality care. Service users valued times when they received emotional support from staff who listened to them and came across as caring. There was a wish among some stakeholders for a more holistic approach to care, for example, more support with social issues. This was not observed in the Twitter sample:

They did listen to me. They did understand my predicament. They acknowledged my dilemma about taking a medication.

CORE service user

Crisis team advising me to take a walk in the park is probably the worst thing to say considering I make self-harm plans in the park

Twitter user

Importantly, a significant aspect of care quality that was frequently discussed by service users on Twitter was receiving unhelpful self-management advice from the CRT. This was unique to Twitter: in the CORE data set, stakeholders did not report experiences of feeling dismissed by distraction or self-care advice.

Both data sets identified staff characteristics as a determinant of good quality treatment, with consensus that there is a wide variety of staff expertise within CRTs. As CRT staff work shifts, the extent to which consistent therapeutic relationships are established varies greatly, and continuity of care is a challenge. This was reflected in both data sets, where continuity of staffing was valued and seen as important in CRT care. Personal staff characteristics, such as empathy and compassion, were consistently described in both forms of data as critical ingredients in good CRT support:

He’s had some good, really good staff come to see him, and he’s had some damn awful, pretty diabolical people come to see him, as well.

CORE carer

Shocked by reading the hashtag #crisisteamfail, I have experienced this but I also think some CRT staff are great

Twitter user

#### Strain on Mental Health Services

In our sample, we found that people posted on Twitter about a strain on mental health services due to high demands and a lack of resources. This theme was unique to the Twitter data set and was not discussed in the CORE interviews and focus groups with service users, despite the broad semistructured nature of the interview questions. Some Twitter users directed tweets toward the Twitter accounts of the NHS and UK government. This theme reflects the political nature of Twitter as a social forum in comparison with interviews and focus groups that take place in a private space. This theme was distinct from the theme “the role of CRTs in the care system” in the CORE data set, which described the role of CRTs as gatekeepers for hospital admissions:

NHS mental health crisis services are being stretched to breaking point.

Twitter user

Police are helping people who are self-harming cos the conservatives slashed funding for CRTs.

Twitter user

## Discussion

### Principal Findings

Our findings suggest that the analysis of Twitter data can complement traditional qualitative research methods and expand our understanding of stakeholder views of mental health crisis care delivered by CRTs. Many of the same things feature in what service users and family caregivers describe as valuable components of good quality CRT care in both fora, particularly staff who listen and provide warmth and empathy. We accessed additional insights into the experiences of CRTs from Twitter. Twitter users posted that self-management strategies suggested by CRT staff were frequently experienced as unhelpful and expressed concern about the strain on mental health services. Twitter users with *personality disorder* diagnoses discussed their diagnosis as a barrier to accessing CRT care. More generally negative sentiments were expressed on Twitter compared with *traditional* face-to-face data collection methods. Therefore, it seems likely that more negative experiences of CRT service use and implementation may be missed by relying solely on face-to-face data collection methods.

Twitter differs from traditional qualitative methods in several respects. Interviews and focus groups are often set up as *spaces for reflection*. This is encouraged in numerous ways: at the time of data collection (usually after some time has elapsed since the experience), in the open nature of questioning, and in the way in which interviews are introduced as research where there are no *right* or *wrong* answers [51]. The interviews and focus groups were also structured to some degree by the researcher. Twitter, on the other hand, is an instant form of communication, where individuals often post using mobile phones in the *heat of the moment* [19,52]. Twitter posts also take place outside of research encounters. When considering these very different research contexts, this strengthens our confidence in the congruent themes in our analysis, as these are expressed both inside and outside of the *interview room*.

Twitter is set up as a social media forum, encouraging interactions between users [23,53,54]. There appeared to be a community of service users with similar negative experiences of CRTs in this study, who engaged and supported each other in the *Twittersphere*. For example, interactions about unhelpful self-management advice appear to have turned into a *running joke* in this Twitter community as part of the hashtag *#crisisteamfail*. This is a clear example of how views are constructed and reinforced in the social sphere of Twitter in a way that is very different from how views are expressed in individual face-to-face methods. Analyzing Twitter posts can therefore allow access to how issues and experiences are shared among socially mediated communities of health service users [23,55].

The participant recruitment processes for interviews and focus groups in the CORE study likely introduced some selection bias, as potential participants were identified via CRTs. This necessarily excludes people in mental health crises who could not or did not want to engage with CRTs. As the CRT staff contributed to identifying service users and their family members, they may have been more likely to identify service users who had engaged positively with CRT care (although CORE study researchers took steps to help avoid selection bias by the staff). For example, participants diagnosed with *personality disorders* in the CORE interviews had received a service from the CRT, so the sample was unlikely to include people who felt that they had been denied care. The Twitter analysis may therefore allow research to access a broader range of voices compared with face-to-face qualitative methods alone.

Individuals who are active on Twitter may differ from those who engage in traditional qualitative research and may differ from CRT service users as a whole. A significant proportion of mental health service users (and nonusers) are distrustful of services or may have had negative previous contacts with services, sometimes involving coercion. These individuals may be more inclined to engage in Twitter than in traditional research projects. In addition, given its broad social reach, people who use Twitter may be more likely to engage as advocates and activists, bringing this distinctive point of view to the table. Twitter can be viewed as a naturalistic public setting in which service users can have their voices heard [23], gaining power in this virtual space where they can choose to remain anonymous. Some people may feel more comfortable communicating through text, rather than face-to-face interactions. Twitter users tend to be from younger sectors of the population [18], whereas CRT service users as a whole tend to be middle-aged [32], which may explain some differences between themes identified in this study and highlights that Twitter may reflect younger service users’ experiences of CRTs. By using interviews and focus groups alone, research on CRTs thus far may fail to access the views of less-engaged service users and more critical stakeholders.

### Strengths and Limitations

This is the first study to compare views expressed on Twitter with data collected from interviews and focus groups in the field of mental health research. A broad range of search terms were used, both with and without *hashtags*, which link tweets together. By conducting the search directly from Twitter, we avoided bias that would have been introduced if we used software such as NCapture [56]. The benefits of including lived experience perspectives in analyzing qualitative data in mental health research to help provide a more complete understanding of the data [57] are equally relevant for research involving Twitter. Collaborative coding in this study enhanced the validity of qualitative analysis: the research team included key stakeholder perspectives and experiences of using, providing, and researching mental health services.

This study has some limitations. The search strategy meant that the demographics of tweeters could not be retrieved. Tweets were accessed through Twitter’s advanced search function, which uses Twitter’s application programming interface. This means that the search output is determined in part by Twitter’s programming. Nevertheless, research suggests that the application programming interface method still retrieves a large proportion of tweets available in the public domain [58]. The search terms used in this study were inclusive, but there will inevitably be some relevant tweets that were missed. The study design meant that we were unable to conduct any causal analysis to explain the differences between the themes in both data sets. Finally, tweets are by nature short and may therefore lack the depth, nuance, or complexity of other narrative forms. There is some room for misinterpretation where there is a lack of context and a lack of opportunity to explore the meaning of tweets further [5]. Collaborative coding and meetings with a wider research team aimed to resolve the uncertainties as much as possible.

### Implications for Clinical Practice and Future Research

The refutational synthesis found that there are some overall congruent messages about what is considered important in CRT practice; however, there were differing views about how far this is being achieved and some themes emerged from only one set of analyses. Our confidence in CORE study findings [38] regarding critical ingredients of good CRT implementation—including good access, responsiveness, and consistent and caring staff—is strengthened by the present findings. This is consistent with previous qualitative studies that identified similar critical ingredients of good CRT services [35]. To our knowledge, our study is the first to examine stakeholder perspectives of CRTs using social media data. The stark comparison between service user experiences accessed using Twitter or face-to-face methods shows that we should approach either data source with caution as a gauge of overall service user experience. This reinforces the need for better and more coordinated routine collection and analysis of patient-reported experience measures in mental health services and highlights the virtues of triangulating different sources in qualitative research [41].

Our analysis suggests that CRTs need to develop new approaches when supporting individuals with self-management advice and that CRT staff should avoid giving general self-care advice, such as going for walks. This is likely to apply to other clinical contexts in mental health care. Instead of self-care advice, self-management strategies should be tailored toward relapse prevention and recovery goals [59]. For example, a randomized controlled trial demonstrated the benefits of having a peer support worker promote self-management strategies for those recently discharged from a CRT [60]. Although research has shown that mental health professionals have mixed opinions about using Twitter in their professional life [2], we argue that Twitter may be a helpful source of viewpoints for service improvement initiatives [1,16]. However, future work is needed to investigate whether Twitter is representative enough to provide real-time feedback on mental health services.

Researchers may find it beneficial to engage proactively with Twitter to gain a broad range of perspectives. This is particularly the case when research questions seek to include those who have difficulty engaging or have disengaged from services or where studies have difficulty recruiting participants for lengthy interviews and focus groups. Future research using Twitter data may use natural language processing techniques to examine the broad content of Twitter posts. It would also be interesting to track trends and changes over time. A limitation of this study was that we could not conduct any causal analysis, and future research may explicitly ask CRT stakeholders to reflect on and explain differences between themes in the Twitter and interview data sets. The themes identified in this study demonstrate that more research is needed on how best to optimize crisis care for people who have been diagnosed with a *personality disorder*, as our Twitter analysis showed that this group often felt dismissed, neglected, and stigmatized by services. Further explorations are desirable to examine the potential of Twitter and other social media platforms in mental health research. We hope that there will be developments in evidence-based best practice for conducting qualitative research using Twitter data and for combining social media analysis with standard interviews.

### Conclusions

Twitter users provided unique perspectives on CRT implementation, including the importance of the type of advice offered to help manage crises and the perceived limitations of CRT services. Research on social media content adds complexity to our understanding of phenomena and can complement traditional or face-to-face research methods in health care research by allowing the voices of people who may be more critical of services to be heard.

## Abbreviations

CORE: Crisis Team Optimization and Relapse Prevention
CRT: crisis resolution team
NHS: National Health Service
